# Association between ultra-processed food consumption and risk of breast cancer: a systematic review and dose-response meta-analysis of observational studies

**DOI:** 10.3389/fnut.2023.1250361

**Published:** 2023-09-04

**Authors:** Long Shu, Xiaoyan Zhang, Qin Zhu, Xiaoling Lv, Caijuan Si

**Affiliations:** ^1^Department of Nutrition, Zhejiang Hospital, Hangzhou, China; ^2^Department of Digestion, Zhejiang Hospital, Hangzhou, China; ^3^Department of Geriatrics, Zhejiang Hospital, Hangzhou, China; ^4^Zhejiang Provincial Key Lab of Geriatrics, Geriatrics Institute of Zhejiang Province, Hangzhou, China

**Keywords:** ultra-processed food, nova, breast cancer, meta-analysis, systematic review

## Abstract

**Background:**

Some epidemiological studies have examined the association between consumption of ultra-processed food (UPF) and the risk of breast cancer. However, the results were inconsistent. Therefore, we carried out a systematic review and dose-response meta-analysis to examine whether an association exists between high consumption of UPF and breast cancer risk.

**Methods:**

PubMed/MEDLINE, ISI Web of Science, EBSCO and CNKI databases were systematically searched from inception to May 2023. The summary relative risks (RRs) and 95% confidence intervals (CIs) associated with UPF consumption and breast cancer were calculated using a random-effects model (DerSimonian-Laird method). Heterogeneity between included studies was examined using the Cochran’s *Q* test and I-square (*I*^2^) statistics. Publication bias was studied by visual inspection of funnel plot asymmetry and Begg’s and Egger’s tests.

**Results:**

Overall, six articles involving 462,292 participants, were eligible to be included in this study. Compared to the lowest consumption, highest consumption of UPF was related to a higher risk of breast cancer (RR = 1.10; 95%CI: 1.00–1.22, *p* = 0.056). Besides, the linear dose–response analysis showed that each 10% increment in UPF consumption was related to a 5% higher risk of breast cancer (RR = 1.05; 95%CI: 1.00–1.10, *p* = 0.048). Subgroup analyses suggested that UPF consumption was positively associated with breast cancer risk in case-control studies (RR = 1.13; 95%CI: 1.01–1.26, *p* = 0.028). Additionally, there was also a significant positive association between UPF consumption and breast cancer risk in the subgroup with sample size<5,000(RR = 1.17; 95%CI: 1.02–1.35, *p* = 0.028).

**Conclusion:**

Our results indicate that higher consumption of UPF is slightly related to a higher risk of breast cancer. Further studies in particular of large prospective cohort studies are warranted to confirm these results.

## Introduction

Cancer ranks as a leading cause of death worldwide, with an estimated 19.3 million new cases and 10.0 million deaths diagnosed in 2020 (1). Breast cancer is one of the most commonly cancers in women, and its incidence continues to rise (2). According to the International Agency for Research on Cancer (IARC), breast cancer has overtaken lung cancer as the leading cause of cancer incidence worldwide by 2020, with an estimated 2.3 million new cases, accounting for 11.7% of all cancer cases (1). Multiple risk factors contribute to an increased risk of breast cancer including alcohol consumption, obesity, a sedentary lifestyle, family history of cancer, menstrual and reproductive history, exogenous hormone intake and never giving birth or breastfeeding have already been identified (3). Apart of these risk factors, dietary factors have also been recognized as an important and modifiable risk factor for the primary prevention of breast cancer (4).

In recent decades, a substantial amount of epidemiological studies has explored the relationship between diet and breast cancer risk (5–8). However, these studies have mainly focused on the effects of individual foods (5), nutrients (7), or dietary patterns (6, 8). At the same time, the World Cancer Research Fund (WCRF) report in 2007 concluded that high alcohol consumption may increase the risk of breast cancer (9). Over the last decade, diets in several high-income countries have shifted toward a dramatic increase in the consumption of ultra-processed foods (UPF), which are typically ready-to-eat, hyper-palatable, cheap and high in energy density, added sugars, salt, saturated and trans- fats, as well as low in dietary fiber, protein, vitamins and micro-nutrients (10, 11). In 2009, the NOVA food classification system was proposed by Brazilian researchers to evaluate foods and beverages consumption based on the nature, extent and purpose of food processing (12). Based on this system, foods and food products are divided into four different groups, including unprocessed and minimally processed food, processed culinary ingredients, processed foods and UPFs (13). Currently, the global consumption of UPF has been rising rapidly, and the United Kingdom and United States are leading consumers with UPF exceeding 50% of daily calorie intake (10, 14). In view of this, considerable attentions have been paid for studying the impact of high UPF consumption on various adverse health outcomes, e.g., overweight/obesity, type 2 diabetes and cancers. Numerous studies have attempted to investigate the relationship between consumption of UPF and risks of obesity, type 2 diabetes, cardiovascular disease and all-cause mortality (11, 15–17). Nevertheless, relatively little is known regarding the relationship between the degree of food processing and risk of common cancers. Until 2018, Fiolet et al. published the first large prospective study of UPF consumption and the risk of cancer based on the French NutriNet-Santé cohort (18). Since then, a growing body of evidence shows that UPF consumption is closely linked to common cancers, such as colorectal, breast and prostate cancers (18–20). Up to date, several epidemiological studies have focused on the relationship between the entire of UPF consumption and risk of breast cancer (18, 20–24). However, the results have been inconsistent. Two previous studies have suggested that UPF consumption was associated with breast cancer (18, 23), while other studies showed no statistical associations (20–22, 24). For example, in the NutriNet-Santé cohort study, including 104,980 participants followed in France, Fiolet and colleagues reported that a 10% increase in the proportion of UPF in the diet was significantly associated with an 11% increased risk of breast cancer (RR = 1.11, 95%CI: 1.01–1.22) (18). Contradictory to the above finding, a recent case-control study performed in South African found that higher consumption of UPF had no significant effect on breast cancer risk (OR = 1.03; 95%CI: 0.72–1.45) (24). To the best of our knowledge, Isaksen et al., recently published a meta-analysis of 11 observational studies (8 retrospective case-control studies and 3 prospective cohorts) evaluating the association between consumption of UPF and cancer risk (25), but this meta-analysis only included three studies reporting the risk of breast cancer. In addition, the results of the above-mentioned meta-analysis only reviewed the studies on UPF consumption and cancer risk without quantitative analysis. The authors pooled the results for different cancer outcomes (e.g., colorectal, breast, prostate, and pancreatic cancers), and compared cancer risk in the highest versus lowest categories of UPF consumption. Furthermore, no meta-analysis thus far has yet assessed the dose–response association between UPF consumption and breast cancer risk. Therefore, to evaluate the impact of UPF consumption on breast cancer, we carried out this comprehensive systematic review and meta-analysis to summarize the evidence from observational studies published from inception to May 2023.

## Methods

The current study was performed according to the Preferred Reporting Items for Systematic Reviews and Meta-Analyses: the PRISMA statement (26).

### Search strategy

We carried out a systematic search and literature review using the following electronic databases: PubMed/MEDLINE, ISI Web of Science, EBSCO and CNKI to identify relevant articles. Databases were from inception up to May 2023, and the search was restricted to human studies. In addition, no restrictions for publication date or language were used. The following keywords or phrases, including those from the medical subject headings (MeSH) and non-MeSH terms, were utilized in this search: (“fast food” OR “processed meat” OR “processed food” OR “ultra-processed food” OR “hamburger” OR “salami” OR “bacon” OR “sausage” OR “luncheon meats”) AND (“breast cancer” OR “breast neoplasms” OR “breast adenoma” OR “breast carcinoma” OR “breast tumor”). The list of references obtained from the retrieved articles and systematic reviews was further manually retrieved into other relevant studies. This search strategy was performed by two of all authors (L.S and X.-L.L). Our selection criteria was based on the PICOS (e.g., participant, intervention/exposure, comparison, outcome, and study design) framework, as shown in Table 1.

**Table 1:** PICOS criteria for inclusion and exclusion of studies.

Population	Adults
Exposure	Ultra-processed food consumption
Comparison	Highest vs. lowest categories of exposure and each 10% increase in exposure
Outcomes	Breast cancer
Study design	Case–control or cohort studies

PICOS, participant, intervention (exposure), comparison, outcome, and study design.

### Study selection

Teams of two authors (LS and XL) independently searched each article from the published literature, and consulted a third author (QZ) to resolve any discrepancies. After selecting the title and abstract of article, the full-text versions of articles were reviewed according to the inclusion and exclusion criteria of this systematic review and meta-analysis. Studies were included in this meta-analysis if they met each of the following criteria: (1) observational studies (e.g., cohort, case-control or cross-sectional studies) performed in adult population (aged ≥18 years); (2) considered UPF based on the NOVA food classification system as the exposure; (3) evaluated the association with breast cancer risk; (4) provided estimates of RRs, HRs, ORs with their corresponding 95% CIs; (5) If the data in published studies were ambiguous or missing, corresponding authors would be contacted for key information by email. In addition, studies were excluded if they fulfilled one of the following criteria: (1) unrelated articles; (2) non-observational studies, e.g., reviews or conference letters; (3) animal studies or *in vitro* studies; (4) studies not reported as HRs, RRs or ORs with 95%CIs; (5) UPF consumption was not assessed using the NOVA food classification system. When the same study published more than one article, we selected the newest publication with the largest number of cases.

### Data extraction

Data were extracted by two independent authors (XL and XZ) from all included studies, including first author’s last name, publication year, study design, study area, sample size, number of breast cancer cases, mean age, duration of follow-up, method of UPF assessment, and confounding factors used for adjustments in the multivariate analysis. Any differences and disagreements regarding data extraction were resolved by consensus or discussion with the third author (QZ).

### Definition of ultra-processed food

According to the NOVA food classification system, a diet rich in UPF is characterized by high intakes of foods made up entirely or mostly from unhealthy components, which typically have high energy density, high amounts of fats, added sugar, and low amounts of fiber, minerals and vitamins (27). Examples of UPF in the included studies are shown in Supplementary Table S1.

### Quality assessment

The two authors (LS and CS) separately evaluated the overall quality of the included studies using the Newcastle-Ottawa Scale (NOS), which was adopted for case- control and cohort studies (28).This NOS consists of eight questions that assess quality in three broad domains: selection of participants (maximum of 4 stars), comparability of the groups (maximum of 2 stars), and outcome/exposure assessment (maximum of 3 stars). Thus, the total NOS score ranged from 0 to 9. Finally, those studies with NOS scores ≥7 points were considered to be of high quality (29). Any disagreements between the two authors were resolved by the third author (LS) to reach a consensus.

### Statistical analysis

In this meta-analysis, we used RRs and 95%CIs as the effect sizes for main analyses. The HRs reported in the original studies were considered equivalent to the RRs (30). In our analyses, ORs were converted into RRs using the formula: RR = OR/[(1−P_0_) + (P_0_*OR)], in which P_0_ indicates the incidence of the outcome of interest in the non-exposed group (31). First, we carried out a pairwise meta-analysis by pooled the RRs and 95% CIs of the highest versus lowest categories of UPF consumption in relation to the risk of breast cancer. Heterogeneity between studies was tested using the Cochran’s *Q* test and and *I*^2^ statistic. If *p* values of Cochran’s *Q*-test ≤0.10 or *I*^2^ ≥ 50% indicated an absence of heterogeneity among studies, and a random-effects model (DerSimonnian and Laird method) was used (32). When significant heterogeneity was observed, sensitivity and subgroup analyses would be performed to further explore the cause of the heterogeneity. In the present meta-analysis, subgroup analyses were carried out based on study design (cohort/case-control studies), outcomes (pre-menopausal/post-menopausal breast cancer), study area (developing/ developed countries), sample size (<5,000/>5,000), exposure assessment(FFQ/24 h dietary recall), and alcohol intake (adjusted/unadjusted). Sensitivity analysis was carried out to clarify whether the summary effect size was robust or sensitive to the influence of a particular study. Publication bias was evaluated by the visual inspection of funnel plots, formal testing by the Egger’s regression asymmetry and Begg’s rank correlation tests (33). If publication bias was observed, the trim and fill method was utilized to re-calculate the pooled effect sizes (34). Second, according to the method introduced by Greenland and Longnecker, we performed a dose-response meta-analysis to estimate the RRs for every 10% increase in UPF consumption (35). Finally, we carried out a one-stage linear mixed-effects meta-analysis to model the dose-response relationship (36). All statistical analyses were carried out using STATA version 12.0 (StataCorp, College Station, TX, United States), with a two-tailed *p*-value ≤0.05 showing statistical significance.

## Results

**Figure 1:**
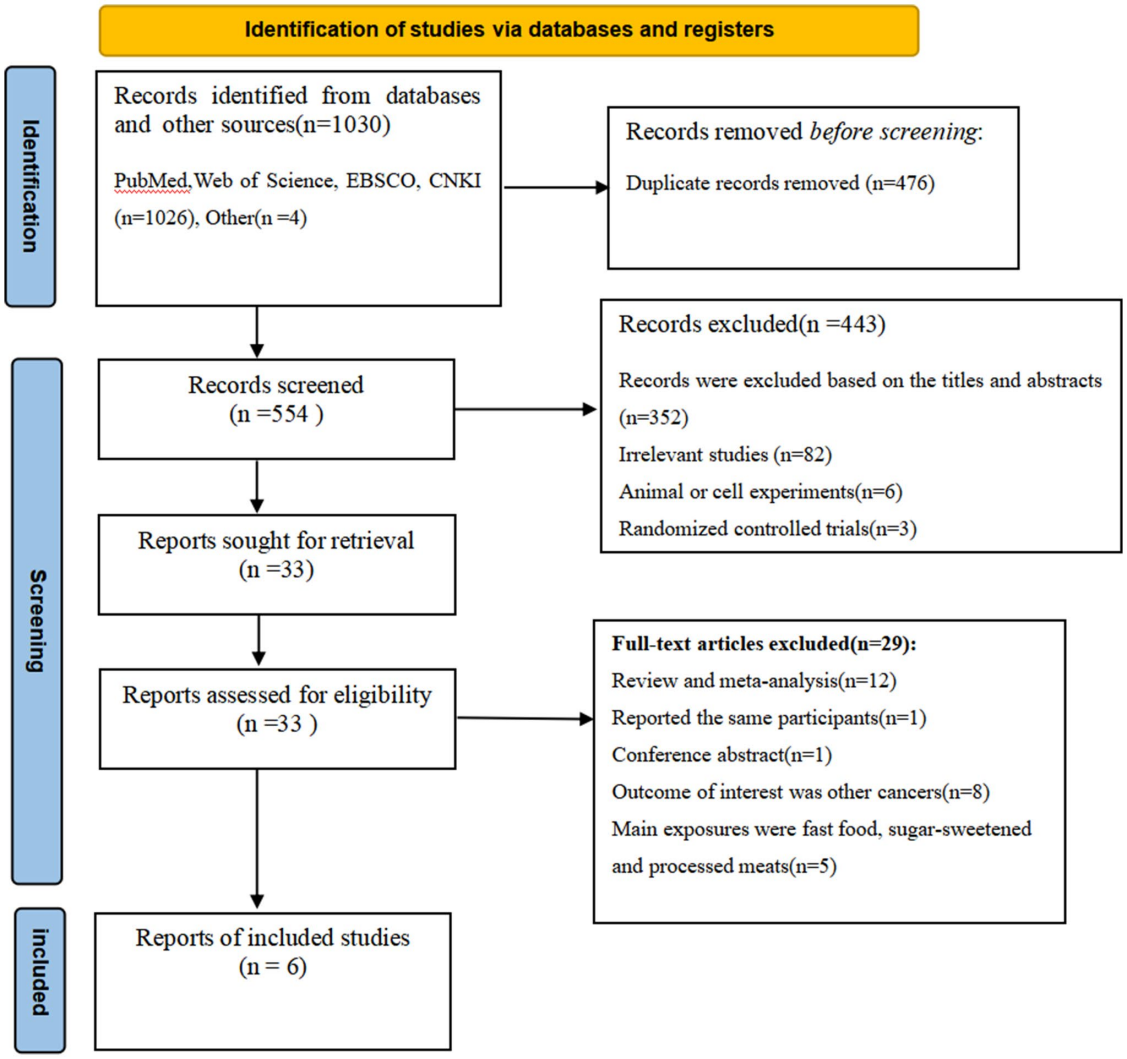
Flow chart of the process of the study selection.

Figure 1 shows the flowchart of the selection of the articles. We identified 1,030 articles through database searches and reference lists. After the removal of duplicates, 476 articles remained. Whereafter, 443 articles were excluded based on the assessment of titles and abstracts and the inclusion criteria. The remaining 33 full-text articles were independently reviewed in details by two authors and 27 articles were excluded for the following reasons: conference abstract (*n* = 1), reviews or meta- analyses (*n* = 12), the main exposures were individual foods, such as fast food, sugar-sweetened beverages and processed meats (*n* = 5), outcomes were other cancers (*n* = 8), and the same population was reported (*n* = 1). Finally, six articles were included in the final analysis.

### Characteristics of the included studies

The characteristics of all the included studies are shown in Table 2. A total of six articles, including 462,292 participants and 18,673 breast cancer cases were included in this study. Of the six studies included, three were cohort studies (18, 21, 22), and the other three were case-control studies (20, 23, 24). All of these included studies were published between 2018 and 2023. The mean follow-up time for prospective cohort studies ranged from 8 to 14.1 years. One of the included studies were conducted in France (18), one in Spain (20), one in the United kingdom (21), one in European countries (22), one in Latin American countries(Chile, Colombia, Costa Rica and Mexico) (23), and one in South African (24). Sample size ranged from 792 to 197,426. Four of the included studies used FFQ to collect dietary intake data (20, 22–24), and other two studies used 24 h dietary recalls (18, 21). All of the included studies classified UPF according to the NOVA food classification system (18, 20–24). Finally, all of the included studies were of high-quality according to NOS scores (18, 20–24).

**Table 2:** Characteristics of included studies on the relationship between UPF consumption and risk of breast cancer.

Studies	Location	Study design	Total number of participants	Age	Exposure assessment	Adjustment or matched for in analyses	Outcomes
Fiolet et al. (18)	France	Cohort	82,159 (739 cases)	≥18 years	24 h dietary recall	Age (timescale), sex, energy intake without alcohol, number of 24 h dietary records, smoking status, educational level, physical activity, height, body mass index, alcohol intake, and family history of cancers, menopausal status, hormonal treatment for menopause, oral contraception, number of children and Western dietary pattern (derived by factor analysis).	Highest versus lowest categories of UPF consumption (HR = 1.14,95% CI: 0.91, 1.44); Hazard ratio for increase of 10% in proportion of ultra-processed food intake in diet (HR = 1.11, 95% CI:1.02,1.21)
Romaguera et al. (20)	Spain	Case-control	1,486 cases 1,652 controls	20–85 years	FFQ	Age, study area, educational level, body mass index, physical activity, smoking, hormone replacement therapy use, oral contraceptive use, family history of breast cancer, age at menarche, age at first pregnancy, number of children, menopausal status, total energy intake, and ethanol intake.	Increment of 10% of UPF in the diet increases the risk of breast cancer (OR = 1.07; 95%CI:1.00,1.15); Highest vs. lowest categories of UPF consumption (OR = 1.24, 95% CI: 1.03, 1.49).
Chang et al. (21)	United kingdom	Cohort	197,426 (3,030 cases)	40–69 years	24 h dietary recall	Age (underlying timescale), ethnicity, smoking status, physical activity level, average household income, highest educational attainment, alcohol intake, body mass index, total daily energy intake, and stratified by sex, height, family history of cancer, index of multiple deprivation quintile, geographical region, baseline menopausal status, use of oral contraceptives, use of hormone replacement therapy, and parity.	Per 10% increment in UPF intake (HR = 1.16, 95%CI:1.02, 1.32); Highest vs. lowest categories of UPF consumption (HR = 1.62,95% CI: 0.98, 2.68).
Kliemann et al. (22)	European countries	Cohort	450,111 (2,223 cases)	Mean age: 51 years	FFQ	Age at recruitment (in 1 year categories), center sex, smoking status and intensity, educational level, physical activity, height, diabetes, BMI, Mediterranean diet, alcohol intake, total energy intake, and total fat, sodium, and carbohydrate intakes at recruitment.	Highest vs. lowest categories of UPF consumption (HR = 0.99,95% CI: 0.96, 1.02); Per 10% increment in UPF intake (HR = 1.00, 95%CI:0.96, 1.04).
Romieu et al. (23)	Latin America	Case-control	525 cases 525 controls	20–45 years	FFQ	Age (±3 years), city district of residence and health insurance institution and adjusted for education, (≤primary/secondary/> secondary), moderate intensity physical activity (continuous), number of full-term pregnancies (continuous), age at first full-term pregnancy (nulliparous/<20; 20–25; ≥25), breast feeding ever (yes/no), BMI (continuous), total energy intake (continuous), energy intake from the other NOVA groups (NOVA 1, NOVA 2, NOVA 3 added simultaneously in the model)	Highest tertile of UPF consumption had 93% higher risk of breast cancer (OR = 1.93,95% CI: 1.11,3.35)
Jacobs et al. (24)	South Africa	Case-control	396 cases 396 controls	≥18 years	FFQ	Individual income per month, ethnicity, physical activity, waist circumference (not adjusted for waist circumference when stratified by obesity status) and menopausal status (not adjusted for menopause when stratified by menopausal status)	Highest tertile vs. lowest tertile of UPF consumption (OR = 1.03,95% CI: 0.72, 1.45).

BMI, Body mass index; CI, Confidence interval; FFQ, Food frequency questionnaire; HR, Hazard ratio; OR, Odds ratio; UPF, Ultra-processed food.

### Ultra-processed food consumption and breast cancer risk

**Figure 2:**
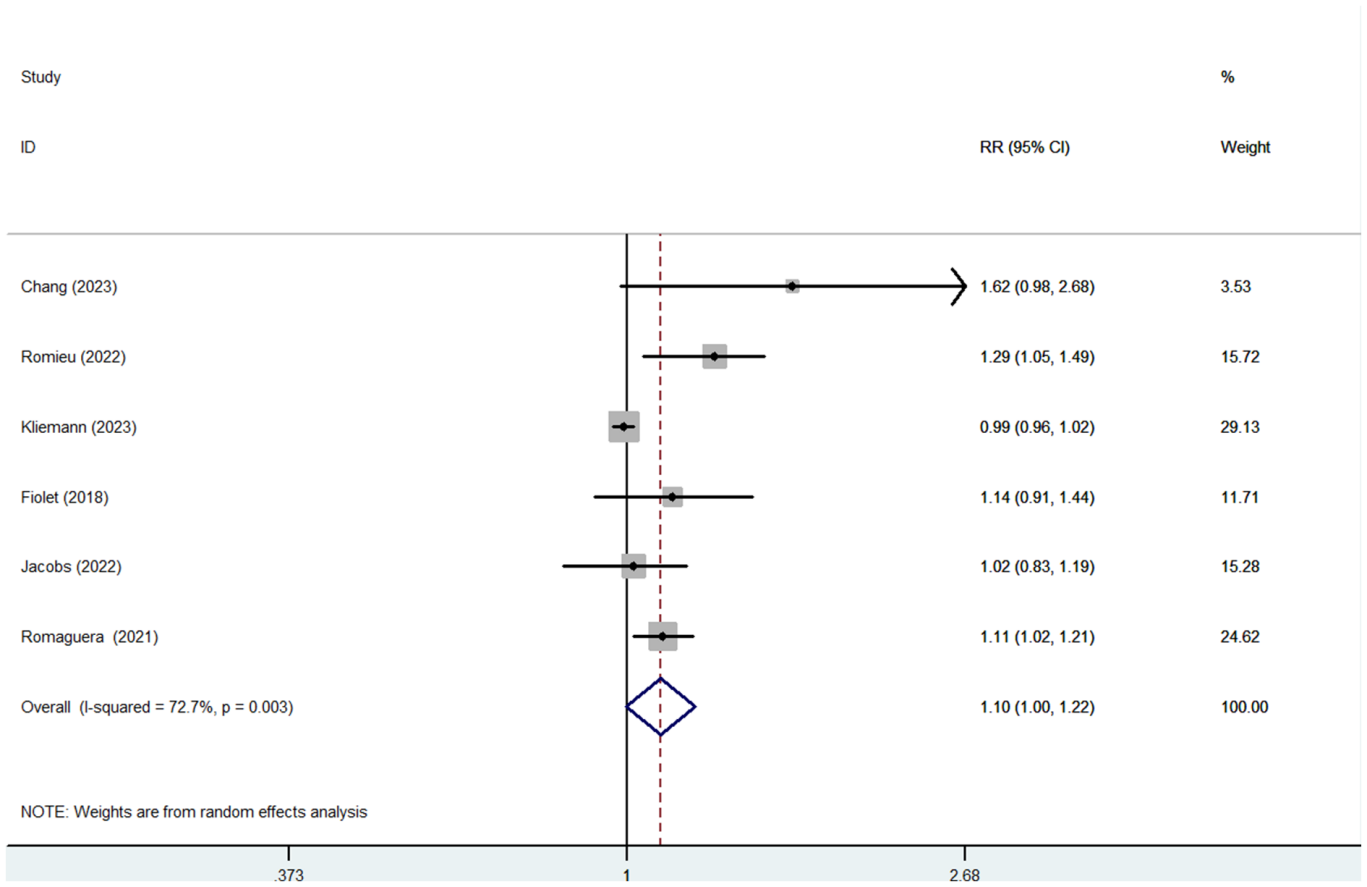
Forest plot of the association between consumption of UPF and breast cancer risk.

**Figure 3:**
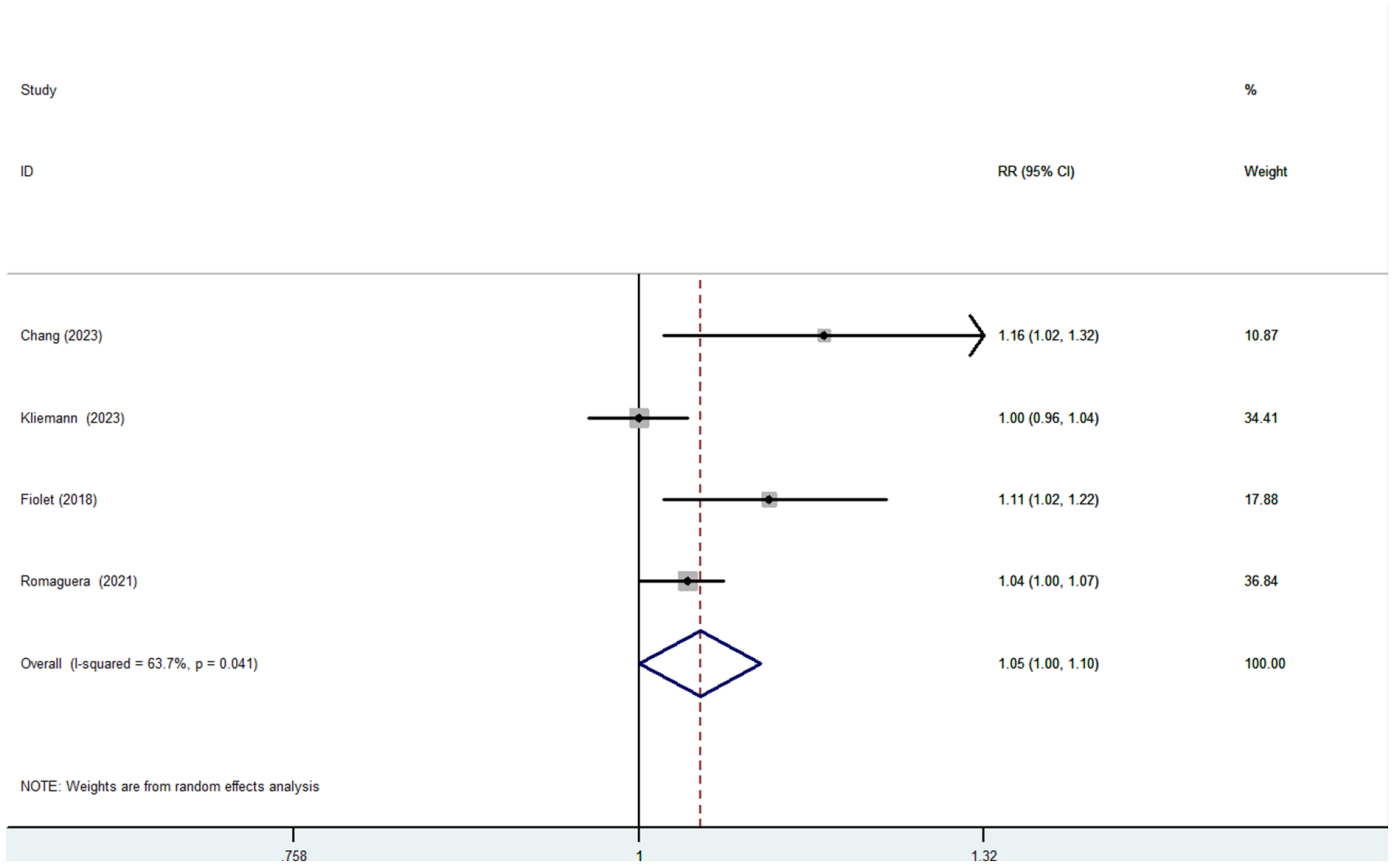
Forest plot of the association between each 10% increase in UPF consumption and breast cancer risk.

**Figure 4:**
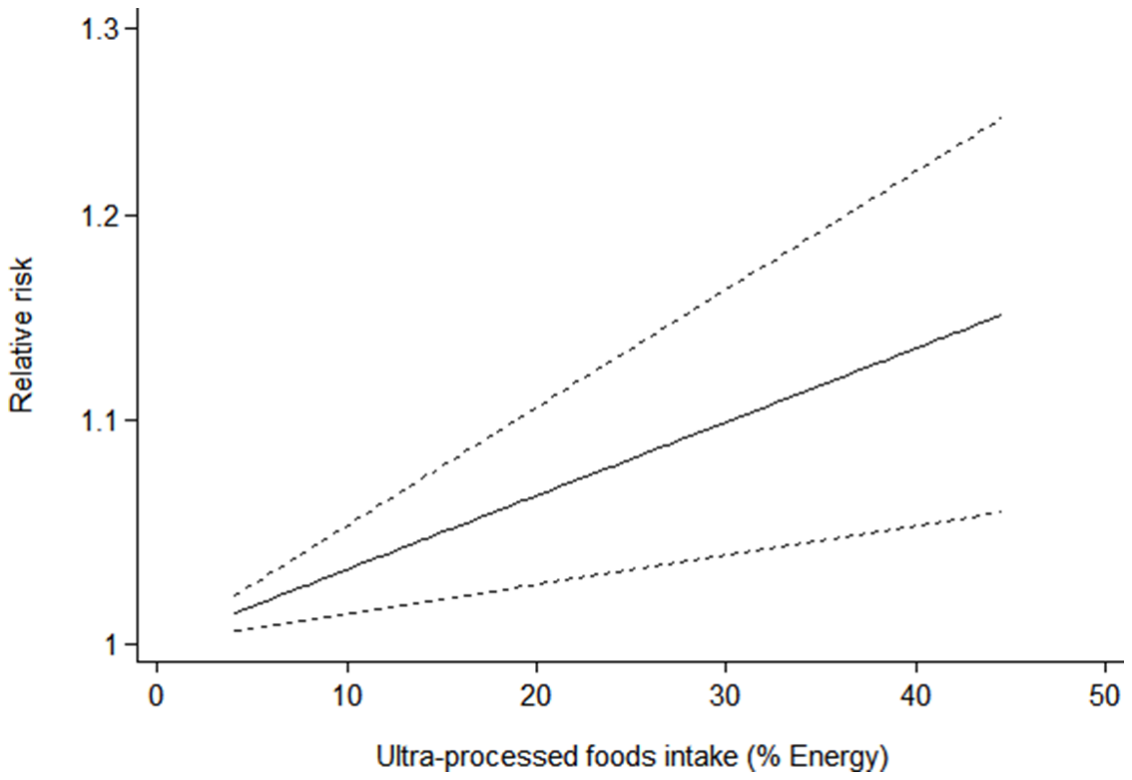
Dose-response association between UPF consumption and breast cancer in the analysis of all included studies.

**Figure 5:**
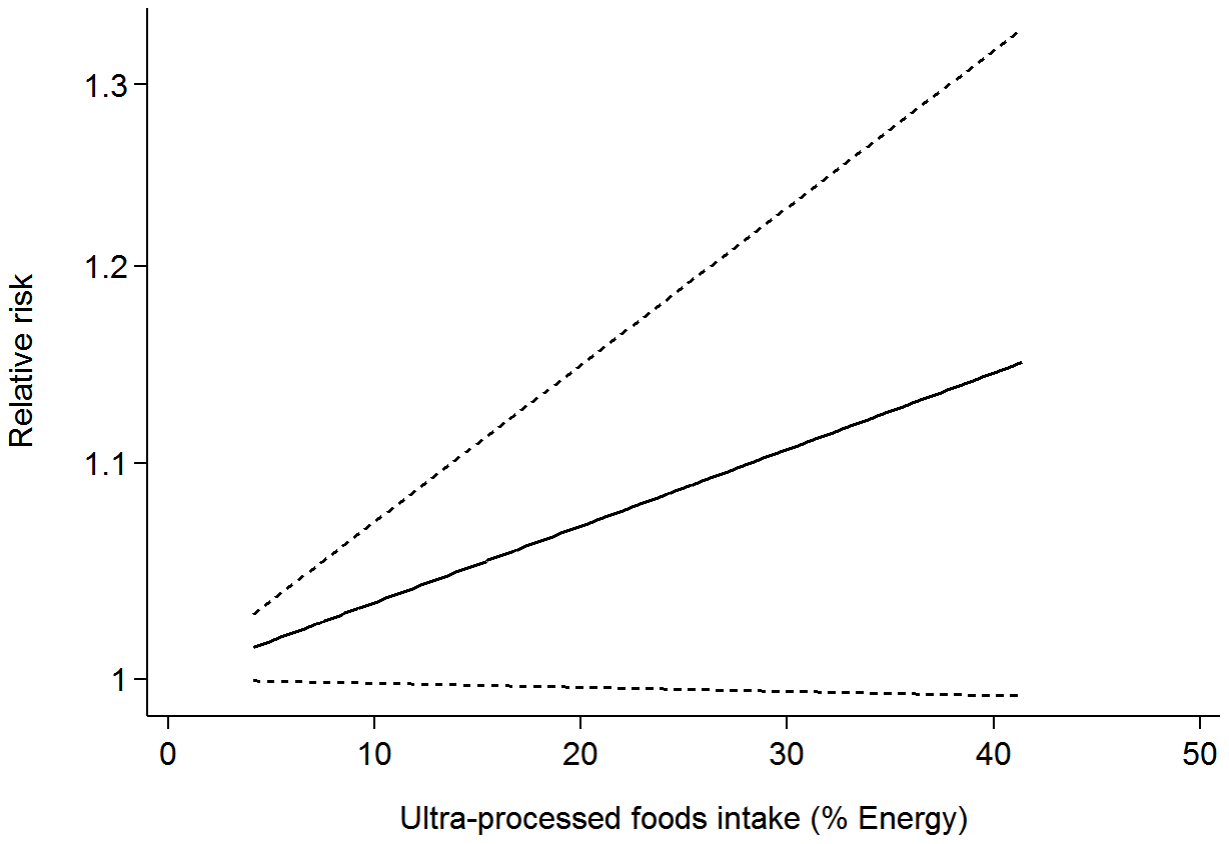
Dose-response association between UPF consumption and risk of premenopausal breast cancer.

**Figure 6:**
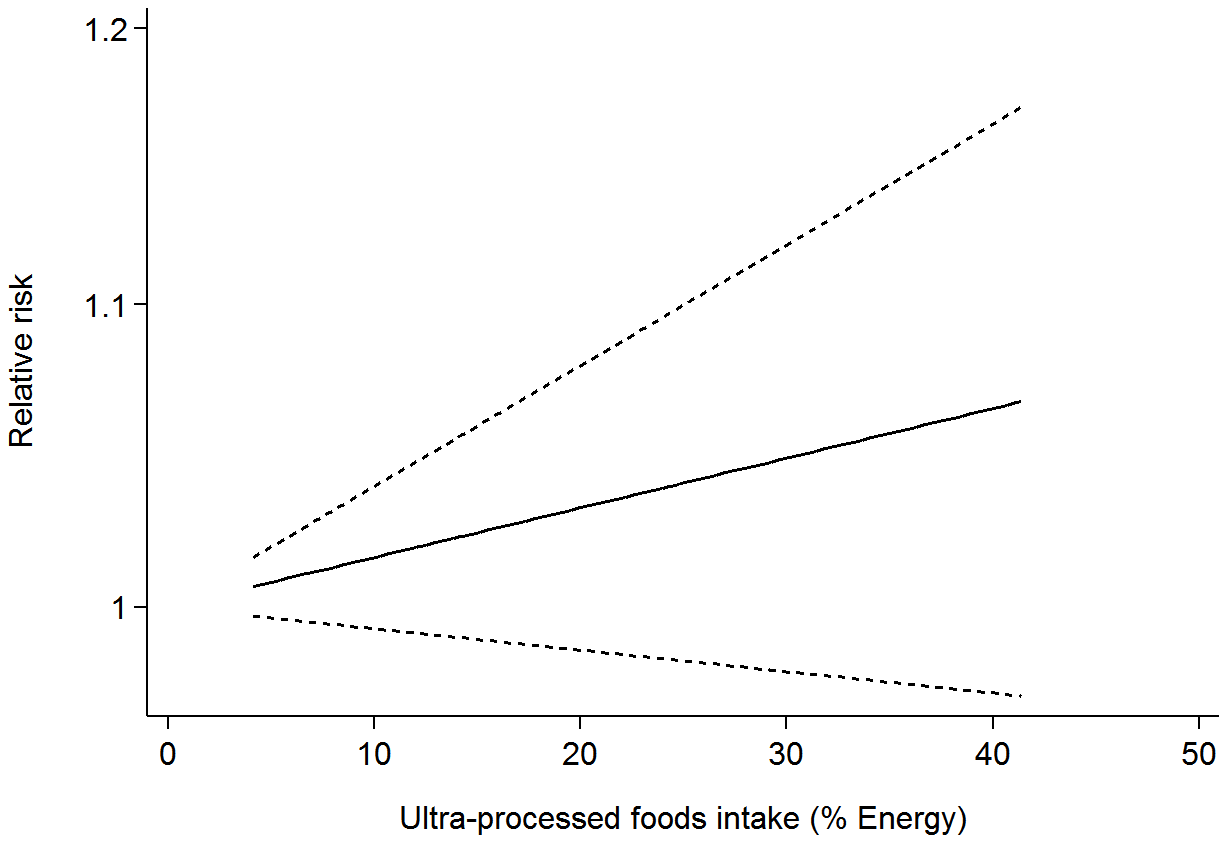
Dose-response association between UPF consumption and risk of postmenopausal breast cancer.

**Figure 7:**
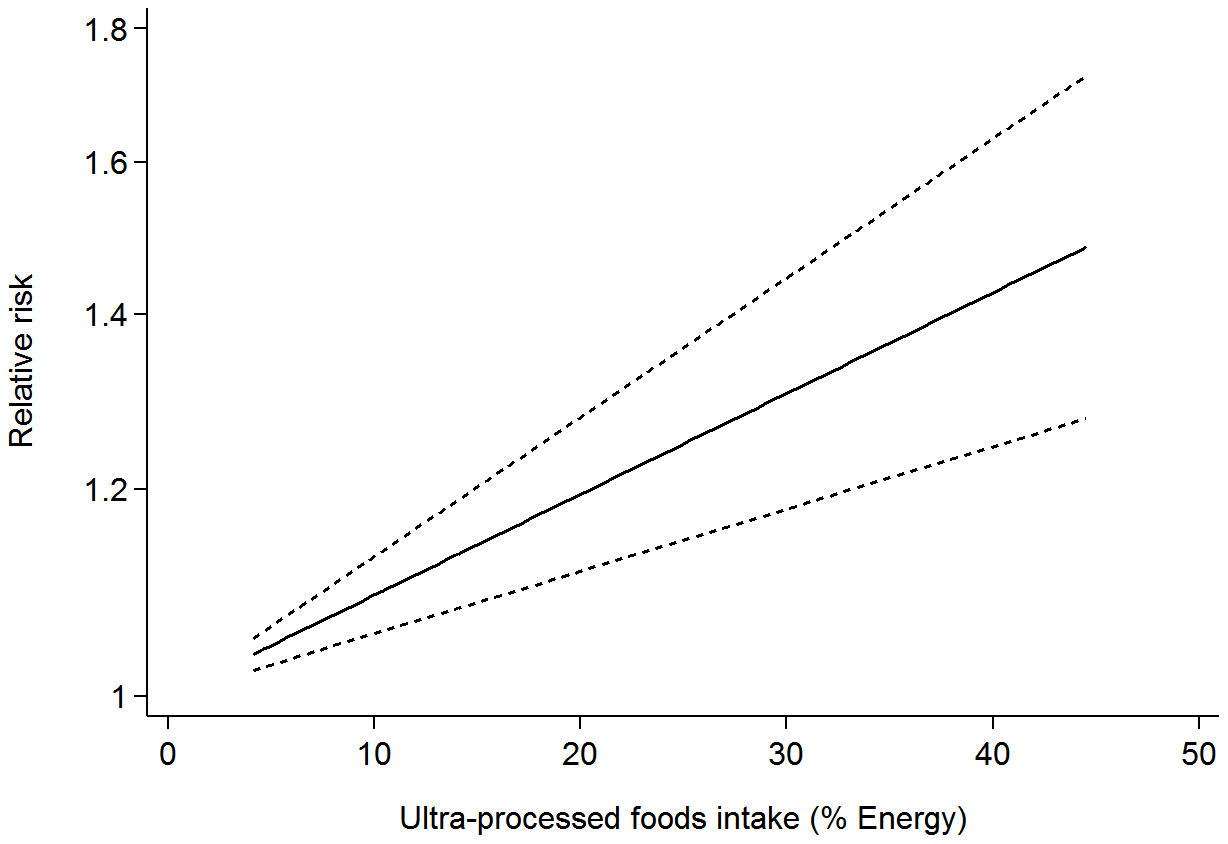
Dose-response association between UPF consumption and hormone receptor positive breast cancer in the analysis of three case-control studies.

Six articles comprising 462,292 participants, were included to assess the link between UPF consumption and risk of breast cancer in this meta-analysis. Figure 2 showed the evidence of an increased risk of breast cancer in the highest category compared with the lowest category of UPF consumption (RR = 1.10; 95%CI: 1.00–1.22; *p* = 0.056). There was evidence of high heterogeneity between studies (*I*^2^ = 72.7%, *p* = 0.003). Figure 3 showed that each 10% increase in UPF consumption was related to a 5% higher risk of breast cancer (RR = 1.05; 95%CI: 1.00–1.10, *I*^2^ = 63.7%; *p* = 0.048). Besides, dose-response associations were presented in Figures 4–7. Figure 4 showed a positive linear relationship between consumption of UPF and risk of breast cancer in the analysis of all included studies (*P*_nonlinearity_ = 0.651, *P*_dose–response_ = 0.001). In addition, the analysis of five included studies showed the positive linear relationship between UPF consumption and risks of premenopausal breast cancer (*P*_nonlinearity_ = 0.705, *P*_dose–response_ = 0.063; Figure 5), postmenopausal breast cancer (*P*_nonlinearity_ = 0.796 *P*_dose-response_ = 0.907; Figure 6) respectively. The analysis of three case-control studies showed a positive linear association between consumption of UPF and hormone receptor positive breast cancer (*P*_nonlinearity_ = 0.880, *P*_dose-response_ = 0.890; Figure 7).

### Subgroup analyses

Given the high heterogeneity of this study (*I*^2^ = 72.7%; *p* = 0.003), subgroup analyses were performed to discern the potential sources of heterogeneity (Table 3). In our study, subgroup analyses were carried out basing on study design (cohort/case-control studies), outcomes (pre-menopausal/post-menopausal breast cancer), study area (developing/developed countries), sample size (<5,000/>5,000), exposure assessment (FFQ/24 h dietary recall), and alcohol intake (adjusted/unadjusted). For study design, the results indicated a positive relationship between UPF consumption and breast cancer risk in case-control studies (RR = 1.13; 95%CI: 1.01–1.26, *p* = 0.028). But, there was evidence of moderate heterogeneity between studies (*p* = 0.167; *I*^2^ = 44.1%). In contrast, no statistical association was found between UPF consumption and risk of breast cancer in cohort studies (RR = 1.10; 95%CI: 0.90–1.34, *p* = 0.347). Similarly, there was significant heterogeneity (*p* = 0.080; *I*^2^ = 60.5%). In terms of sample size, we found that UPF consumption was positively associated with breast cancer risk in sample size<5,000 (RR = 1.17; 95%CI: 1.02–1.35, *p* = 0.028). However, there is still significant heterogeneity (*p* = 0.130, *I*^2^ = 56.3%). Meanwhile, no significant association was found between UPF consumption and breast cancer risk in the studies with sample size>5,000 (RR = 1.04; 95%CI: 0.93–1.16, *p* = 0.456), and there was moderate heterogeneity (*p* = 0.162, *I*^2^ = 41.6%).

**Table 3:** Subgroup analyses for the association between ultra-processed food consumption and risk of breast cancer.

Study characteristic	Category	No. of studies	RR (95%CI)	*p*-values	Heterogeneity		
					*p*-values for within groups	*I* ^2^ **(%)**	*p*-values for between groups
Study design	Case-control	3	1.13 (1.01–1.26)	0.028	0.167	44.1	0.002
	Cohort	3	1.10 (0.90–1.34)	0.347	0.080	60.5	
Exposure assessment	24 h dietary recall	2	1.26 (0.92–1.72)	0.144	0.35.6	35.6	0.090
	FFQ	4	1.08 (0.97–1.20)	0.155	0.003	78.4	
Outcomes	Pre-menopausal breast cancer	6	1.12 (0.97–1.29)	0.138	0.004	71.0	0.666
	Post-menopausal breast cancer	5	1.03 (0.96–1.11)	0.435	0.156	39.8	
Study area	Developed countries	4	1.08 (0.96–1.20)	0.188	0.013	72.1	0.041
	Developing countries	2	1.15 (0.91–1.45)	0.239	0.067	70.2	
Sample size	<5,000	2	1.17 (1.02–1.35)	0.028	0.130	56.3	0.001
	>5,000	4	1.04 (0.93–1.16)	0.456	0.162	41.6	
Alcohol intake	Adjusted	4	1.08 (0.96–1.20)	0.188	0.013	72.1	0.041
	Unadjusted	2	1.15 (0.91–1.45)	0.239	0.067	70.2	

FFQ, Food frequency questionnaire; RR, relative risk; CI, confidence interval.

### Publication bias

As presented in Supplementary Figure S1, examination of the funnel plot revealed no evidence of asymmetry. Begg’s test for publication bias was not statistically significant (*p* = 0.707). On the contrary, Egger’s test for publication bias was statistically significant (*p* = 0.037). We used the trim and fill method to re-estimate the effect size, which indicated that the degree of asymmetry was low and the overall effect did not change.

### Sensitivity analysis

Based on the results of sensitivity analysis (Supplementary Figure S2), Kliemann et al.’s study exceeded its limits, and might be the source of heterogeneity. When Kliemann et al.’s study was excluded in a replicated analysis (Supplementary Figure S3), results showed a slight increase in the summary RRs regarding the association between UPF consumption and risk of breast cancer (RR = 1.13; 95%CI:1.06–1.21, *p* < 0.0001). Meanwhile, the overall heterogeneity also decreased from 72.7 to 28.4%.

### Quality assessment

Based on NOS, the quality of all included studies is presented in Table 4. When included studies with a NOS score ≥ 7, they would be regarded as high-quality studies (18, 20–24).

**Table 4:** Ultra-processed food consumption and risk of breast cancer: assessment of study quality.

Studies	Selection				Comparability		Outcome			Score
	1	2	3	4	5A	5B	6	7	8	
Cohort										
Fiolet et al. (18)	*	*	*	*	*	*	*	*	*	9
Chang et al. (21)	*	*	*	*	*	*	*	*	*	9
Kliemann et al. (22)	*	*	*	*	*		*	*	*	8
Case-control										
Romaguera et al. (20)	*	*	*		*	*	*	*		7
Romieu et al. (23)	*	*	*		*	*	*	*		7
Jacobs et al. (24)	*	*	*		*	*	*	*		7

*For case-control studies, 1, indicates cases independently validated; 2, cases are representative of population; 3, community controls; 4, controls have no history of breast cancer; 5A, study controls for the most important factor; 5B, study controls for additional factor(s), e.g., cigarette smoking body mass index, total energy intake; 6, ascertainment of exposure by blinded interview or record; 7, same method of ascertainment used for cases and controls; and 8, non response rate the same for cases and controls. For cohort studies, 1 indicates exposed cohort truly representative; 2, non exposed cohort drawn from the same community; 3, ascertainment of exposure by secure record (e.g., surgical records) or structured interview; 4, outcome of interest was not present at start of study; 5A, study controls for the most important factor; 5B, study controls for additional factor(s); 6, assessment of outcome is based on independent blind assessment or record linkage; 7, follow-up long enough (≥5 years) for outcomes to occur; and 8, adequacy of follow up of cohorts (all participants complete follow up or > 90% participants complete follow up).

## Discussion

To our knowledge, no meta-analysis hitherto has comprehensively evaluated the relationship between consumption of UPF and breast cancer risk. This study is the first dose-response meta-analysis to evaluate the relationship between consumption of UPF and the risk of breast cancer. In the present study, we observed that high consumption of UPF was related to an increased risk for breast cancer. Also, an increment of 10% of UPF in diet was related to a 5% higher risk of breast cancer. Nevertheless, our findings should be interpreted with caution due to the high degree of heterogeneity in all included studies. To explore the foregoing heterogeneity, we conducted subgroup analyses based on study design, menopausal status, study area, sample size, exposure assessment and alcohol intake. Moreover, sensitivity analysis showed that excluding Kliemann et al.’s study could slightly modify the summary effect. Our findings are similar to previous studies, i.e., Fiolet et al. (18) and add to the current knowledge that higher consumption of UPF might exert the detrimental effect on breast cancer.

Basing on data from the IARC, 2300,000 new cases of breast cancer and 1,685,000 deaths were estimated to occur in 2020, accounting for 1/4 cancer cases and for 1/6 deaths among women (1). In light of the high prevalence of breast cancer and its increasing burden on public health, there is an urgent need to explore the possible contributors to this cancer. In fact, being a modifiable risk factor, diet has long been considered as a primary strategy for cancer prevention (37). The IARC on cancer has demonstrated that consumption of red and processed meat intake may be potential carcinogens in humans (38). Over the past few decades, diets in some countries have shifted toward increased UPF consumption, which was characterized by an increase in energy density and a decline in nutritional quality (18). Global UPF consumption has reportedly been rising rapidly in middle-and high-income countries, accounting for approximately 25% ~ 60% of total daily energy intake (10, 22, 39). Thus, studying UPF consumption could be useful to be added in dietary guidelines. At the same time, considerable attentions have been paid in the last decade on the potential effects of UPF consumption on major chronic non-communicable diseases, including breast cancer. Up to now, many epidemiological studies have demonstrated that higher UPF intake is closely related to poor health outcomes, such as overweight/obesity, type 2 diabetes, cardiovascular disease and all-cause mortality (11, 15–19). Consistently, several meta- analyses have also assessed the associations between UPF consumption and risks of chronic diseases (11, 15, 17). Previous these systematic review and meta-analyses showed that high UPF consumption could increase the risks for type 2 diabetes, overweight/obesity and all-cause mortality. Still, no previous meta-analysis to date has assessed the dose-response association between consumption of UPF and risk of breast cancer. To better understand the relationship between UPF consumption and breast cancer risk, we carried out this comprehensive systematic review and dose-response meta-analysis to aggregate the results of observational studies published up to May 2023.

### Comparison with epidemiological literature

In this meta-analysis, the results indicated that higher consumption of UPF was positively associated with the risk of breast cancer. Our findings are in accordance with previous meta-analyses reporting that high consumption of specific types of UPF, e.g., processed meat, fast food, sugar-sweetened and artificially sweetened beverages, were associated with an increased risk of breast cancer (40–42). Of particular concern is that the majority of previous meta-analyses only compared breast cancer risk in the highest and lowest categories of specific types of UPF consumption. However, to date, limited studies have investigated the link between the entire category of UPF and breast cancer risk (18, 20–24), and the conclusions of above-mentioned studies are still inconsistent. For instance, in the French NutriNet-Santé cohort, Fiolet and colleagues reported that a 10% increase in the proportion of UPF in the diet was significantly related to a 11% increased risk of breast cancer (RR = 1.11, 95%CI: 1.01–1.22) (18). Additionally, in a multicentric, population based case-control study, Romieu et al., also found that consumption of ultra-processed foods was associated with an elevated risk of breast cancer in young women (OR = 1.93, 95%CI: 1.11–3.35) (23). Contradictory to our findings, in another large cohort of British adults, Chang et al. found that each 10% increment in UPF intake was not related to an increase in breast cancer incidence over a median follow-up time of 9.8 years (RR = 1.00, 95%CI: 0.97–1.03) (21). Likewise, in a multicentric population-based case-control study conducted in Spain, Romaguera et al., failed to find any significant association between consumption of ultra-processed foods and drinks and risk of breast cancer (20). The reasons for these discrepancies in results are difficult to fully elucidate. But, there are several potential differences in assessing UPF consumption, the amount and type of UPF consumed within study population, duration of study follow-up and alcohol intake that may explain part of these discrepant results. First, the amounts and types of UPF intake in different countries may be different. For instance, the prospective EPIC cohort study has reported that UPF contributed a mean of 13.7% to total energy intake in grams in ten European countries (22). In contrast, the South African breast cancer study reported that UPF consumption contributed to 44.8% in cases and 47.9% in controls (24). Second, four of six included studies used FFQs to collect data on UPF consumption (20, 22–24) and remaining two study using a 24 h dietary recalls (18, 21). Third, a longer duration of study follow-up may be needed for the harmful effect of UPF intake to become apparent. Finally, compared to other studies, Romieu et al., did not adjusted alcohol intake in the multivariable model, and observed a strong positive association between UPF consumption and breast cancer risk. Thus, we hypothesized that alcohol consumption was of a high risk for breast cancer. Of particular concern, a recent meta-analysis showed a consistently significant association between UPF intake and risks of overall and several other cancers, including colorectal cancer, breast cancer and pancreatic cancer (25), which is in agreement with our findings. However, as we mentioned before, Isaksen et al.’s meta-analysis has several significant limitations. For example, the authors only included three articles assessing the relationship between UPF consumption and breast cancer, and compared cancer risks in the highest versus lowest categories of UPF consumption. Most importantly, the dose-response relationship between UPF consumption and breast cancer was not performed in their analyses. In comparison, our study included more original articles, and performed the multiple types of meta-analyses (e.g., highest versus lowest categories of UPF consumption, and linear dose-response analysis), thereby providing more accurate and stable evidence. In this study, the dose-response meta-analysis also showed that every 10% increment of UPF intake in daily calorie intake was related to a 5% increased risk of breast cancer. However, it is worth noticing that the number of publications on the association of UPF consumption with breast cancer remains inadequate, and more prospective studies are required to confirm our results.

Although epidemiological evidence on the association of UPF consumption with risk of breast cancer remains inconsistent, some potential mechanisms have been reported to plausibly explain the observed positive relationship. First of all, UPF consumption has been reported to be related to an increased risk of overweight/obesity or abdominal obesity (11), all of which are well-known risk factors for breast cancer (43). Second, high consumption of UPF has also been related to higher glycaemic response and lower satiety effect (44). A previous meta-analysis of 36 cohort studies showed a positive relationship between glycaemic index and glycaemic load and risk of breast cancer (45). Third, the detrimental effect of UPF consumption on breast cancer may be attributed in part to lower consumption of fresh vegetables and fruits, legumes and whole grains. As far as we know, the above-mentioned these foods are good dietary source of dietary fiber. Aune et al. reported that dietary fiber intake was inversely associated with the risk of breast cancer (46). And also, vegetables and fruits are equipped with abundant antioxidants, such as vitamin C and carotenoids. Available evidence has suggested that antioxidants could neutralize reactive oxygen species and prevent free radical damage in the carcinogenic (47). Fourth, it is well-known that carbonated drinks are the major component of UPF. Previous studies have demonstrated that high consumption of sugar-sweetened beverage may increase the risk of breast cancer (48). Fifth, beyond the nutritional aspects, UPF usually contains some food additives that may be involved in progression of breast cancer. For example, titanium dioxide (TiO_2_), a common food additive, is used as a whitener or in packaging that comes in contact with food or beverages to provide a better texture and antimicrobinal properties (18). Notably, the WHO and the IARC have assessed TiO_2_ as “probably carcinogenic to humans” (group 2B) (49). In addition, food processing, particularly high-temperature heating and extruding methods may produce some neoformed contaminants in ultra-processed products, such as acrylamide, which has been classified by IARC as a Group 2A carcinogen (probably carcinogenic to humans) (50). Sixth, the carcinogenicity of UPF consumption may be attributed to mutagenic compounds, e.g., heterocyclic amines, which are by-products of cooking processed meat (a specific type of UPF) at high temperatures (51). Seventh, UPF is usually packaged in the synthetic substances, such as phthalates and bisphenol A. It is now well-established that phthalates are endocrine-disrupting chemicals commonly used in food storage, packaging and contact materials, and that higher concentration of phthalates in urine has been found in participants with higher UPF consumption (52). In a Danish national cohort study, Ahern et al. found that high-level dibutyl phthalate exposure (≥10,000 cumulative mg) was associated with an approximately two-fold increase in the incidence of estrogen receptor-positive breast cancer (53). Also, bisphenol A, another contaminant, has also been judged as “a substance of very high concern” by the European Chemicals Agency (54). A recent case-control study by López-Carrillo et al., showed that urinary urinary free-bisphenol A is positively related to breast cancer (55). Finally, the adverse effect of UPF consumption on breast cancer may be attributed in part to higher alcohol consumption. Recently, in a Mendelian randomization study, Zhou et al. found an observational dose-response relationship between alcohol intake and breast cancer incidence (56). Additionally, alcohol has been classified as carcinogenic to humans by the IARC. All together, previously mentioned these mechanisms may explain the adverse relationship between high consumption of UPF and breast cancer risk.

In our analyses, the results showed the high between-study heterogeneity on the relationship between consumption of UPF and breast cancer risk (*I*^2^ = 72.7%; *p* = 0.003). Herein, we carried out subgroup analyses of study design (cohort/case-control studies), outcomes (pre-menopausal/post-menopausal breast cancer), study area (developing/developed countries), sample size (<5,000/>5,000), exposure assessment (FFQ/24 h dietary recall), and alcohol intake (unadjusted/adjusted) to explore the sources of heterogeneity. Notably, the results demonstrated that significant heterogeneity might be party due to the differences in sample size and study design. After stratification, moderate heterogeneity was observed in the subgroups of case-control studies and sample size<5,000. Despite the exact reasons for this high heterogeneity are unclear, several possible explanations have already been put forward. First, different levels of UPF consumption in the included studies could partly explain the high heterogeneity between studies. Second, because all of the included studies were observational in nature, the results could be affected by residual or unmeasured factors. Furthermore, half of the included studies were case-control designs. Thus, recall and selection biases should not be ignored. Third, in the present meta-analysis, the included studies have classified the consumption of UPF based on different criteria, such as percentage (%) of total energy intake (kcal) or servings/d. This might be partly attributed to the high heterogeneity. Fourth, different adjusted variables were used in the included studies, which could explain the significant heterogeneity. Finally, a high degree of heterogeneity remained in subgroup analyses, suggesting the presence of other unmeasured confounding factors.

### Strengths and limitations

This meta-analysis has some strengths. First, to our knowledge, this is the first dose-response meta-analysis to discuss the relationship between consumption of UPF and the risk of breast cancer. Our findings add to the growing evidence of an adverse effect UPF consumption on breast cancer and help inform public policy for the prevention of breast cancer. Second, we make rigorous article selection based on the pre-determined inclusion criteria, and only studies that followed the characteristics proposed by the NOVA system are included. Third, breast cancer cases were identified through clinicians’ medical records and pathological reports, reducing the risk of misdiagnosis. Fourth, the sufficient number of included studies allowed us to perform subgroup analyses for some important risk factors, e.g., study design and menopausal status. Meanwhile, we also carried out a dose-response analysis to provide more detailed insight into the relationship between consumption of UPF and breast cancer risk. Fifth, the included studies were of high, and the RRs were multivariate and adjusted for a number of known confounding factors. Finally, there were no significant signs of publication bias in the funnel plots, and statistical tests of publication bias, such as Begg’s test were not significant. Despite the aforementioned strengths, some limitations should be acknowledged. First, in the current meta-analysis, half of the included studies were case-control designs. Thus, we cannot rule out whether these findings are susceptible to recall and selection biases. Second, in all of the included studies, UPF consumption was measured using FFQs or 24 h dietary recalls that were not explicitly designed to collect dietary data basing on the NOVA food classification. Hence, this limitation may cause the misclassification, resulting in the under- or over-estimation of UPF consumption. Third, even though some common confounding factors have been adjusted in the analyses, the existence of residual confounding from unmeasured factors cannot be completely excluded because each included study has inconsistent adjustment for potential confounders. Fourth, high level of heterogeneity was observed in this study. While we carried out subgroup and sensitivity analyses to explore potential sources of heterogeneity, we were unable to adequately ascertain and explain the sources of inter-study heterogeneity. Finally, the majority of the included studies were conducted in Western countries, with remaining two studies in Latin America and South Africa, which could compromise the generalization of our findings.

## Conclusion

To conclude, the findings of this meta-analysis showed that high consumption of UPF was related to a small increased risk of breast cancer. Meanwhile, the linear dose- response analysis also demonstrated that each 10% increase in UPF consumption was related to a 5% higher risk of breast cancer. The present study adds valuable evidence to the literature indicating the harmful effect of UPF consumption on breast cancer risk. Also, our findings may also help physicians in clinical practice by provide some evidence about the role of UPF consumption in the primary prevention of breast cancer. However, due to the limited evidence, further research, especially large prospective cohort studies, is needed to corroborate these findings in different countries and regions around the world.

## Data availability statement

The original contributions presented in the study are included in the article/Supplementary material, further inquiries can be directed to the corresponding author.

## Author contributions

LS was responsible for study concept and design, and drafted the manuscript. XL performed the statistical analysis. QZ was responsible for literature search and screening. XZ conducted the data extraction. LS and CS conducted the quality assessment. LS and XL were responsible for analysis and interpretation of the data. CS and QZ critically revised the manuscript for important intellectual content. QZ and XL made a significant contribution to the data collection analysis and funding of this study. All authors have read and agreed to the published version of the manuscript.

## Funding

This research was funded by the National Natural Science Foundation of China (No. 82004040), Medical and Health research fund project of Zhejiang Province (No. 2022KY006), and Traditional Chinese Medicine Research Project of Zhejiang (Nos. 2020ZB009 and 2021ZB010). These funding sources had no role in the design of the study, in the collection, analyses, or interpretation of data, in the writing of the manuscript, or in the decision to publish the results.

## Conflict of interest

The authors declare that the research was conducted in the absence of any commercial or financial relationships that could be construed as a potential conflict of interest.

## Publisher’s note

All claims expressed in this article are solely those of the authors and do not necessarily represent those of their affiliated organizations, or those of the publisher, the editors and the reviewers. Any product that may be evaluated in this article, or claim that may be made by its manufacturer, is not guaranteed or endorsed by the publisher.

## Abbreviations

AICR: American Institute for Cancer Research
CI: Confidence interval
CNKI: China National Knowledge Infrastructure
FFQ: Food frequency questionnaire
HR: Hazard ratio
IARC: International Agency for Research on Cancer
NOS: Newcastle-Ottawa Quality Scale
OR: Odds ratio
RR: Relative risk
SEs: Standard errors
UPF: Ultra-processed food
WCRF: World Cancer Research Fund
