# Supplementary material for: Association between ultra-processed food consumption and risk of breast cancer: a systematic review and dose-response meta-analysis of observational studies
Source: Front Nutr. 2023 Sep 4;10:1250361. doi: 10.3389/fnut.2023.1250361 (PMC10507475; doi:10.3389/fnut.2023.1250361)
Supplement: Supplementary file 1 [file Table_1.doc]

**Table S1. The foods included in the UPF category**

| Author, year | Ultra-processed food assessment method | Examples |
| --- | --- | --- |
| Fiolet et al. 2018(18) | NOVA: a food classification system  based on the extent and purpose of industrial food processing | Carbonated drinks; sweet or savoury packaged snacks; ice-cream, chocolate, candies (confectionery);mass-produced packaged breads and buns; margarines and spreads; industrial cookies (biscuits), pastries,cakes, and cake mixes; breakfast ‘cereals’, ‘cereal’ and ‘energy’ bars; ‘energy’ drinks; flavoured milkdrinks; cocoa drinks; sweet desserts made from fruit with added sugars, artificial flavours and texturizingagents; cooked seasoned vegetables with ready-made sauces; meat and chicken extracts and ‘instant’ sauces; ‘health’ and ‘slimming’ products such as powdered or ‘fortified’ meal and dish substitutes; ready to heat products including pre-prepared pies, pasta and pizza dishes; poultry and fish ‘nuggets’ and ‘sticks’,sausages, burgers, hot dogs, and other reconstituted meat products, and powdered and packaged ‘instant’ soups, noodles and desserts. |
| Romaguera et al. 2021(20) | NOVA food classification | Sweet or savoury packaged snacks, sweetened beverages, and ready to eat foods |
| Chang et al. 2023(21) | NOVA food classification | Soft drinks, mass-produced industrial-processed breads, sweet or savoury packaged snacks, breakfast ‘cereals’, reconstituted meat products and ready-to-eat/heat foods |
| Kliemann et al 2023(22) | NOVA classification system. | Processed meats(e.g.,reconstituted meat products or sausage, ham, and other meat products), carbonated soft drinks, packaged breads and buns, sweet or savoury packaged  snacks, chocolate, and ready-to-eat meals. |
| Romieu et al. 2022(23) | NOVA classification | Carbonated beverages and industrial fruit juices, flavoured yoghurt and dairy products, distilled alcoholic beverages, industrial breads, breakfast cereals, confectionery, package sweet snacks, packaged savoury snacks, cakes and desserts, sausage and reconstituted meat products, ready-to-eat/heat foods, industrial cheese and cheese substitutes, margarine and butter substitutes and other ultra-processed foods (including sauces, gravies, jam, mayonnaise, artificial sweeteners) |
| Jacobs et al.2022(24) | The NOVA system. | Carbonated drinks, industrial pre-packaged fortified* bread and buns, cookies, pastries,  cakes, cake mixes, breakfast cereals, energy bars, margarines and spreads, instants sauces, soup powders, pre-prepared pies, pasta and pizza dishes, reconstituted meat products, sweet and savoury packaged snacks, ice-cream, fruit yogurts, instant desserts and noodles and distilled alcohol products such as whiskey, gin, rum and vodka |
