# Supplementary figures and images for: Association between ultra-processed food consumption and risk of breast cancer: a systematic review and dose-response meta-analysis of observational studies
Source: Front Nutr. 2023 Sep 4;10:1250361. doi: 10.3389/fnut.2023.1250361 (PMC10507475; doi:10.3389/fnut.2023.1250361)

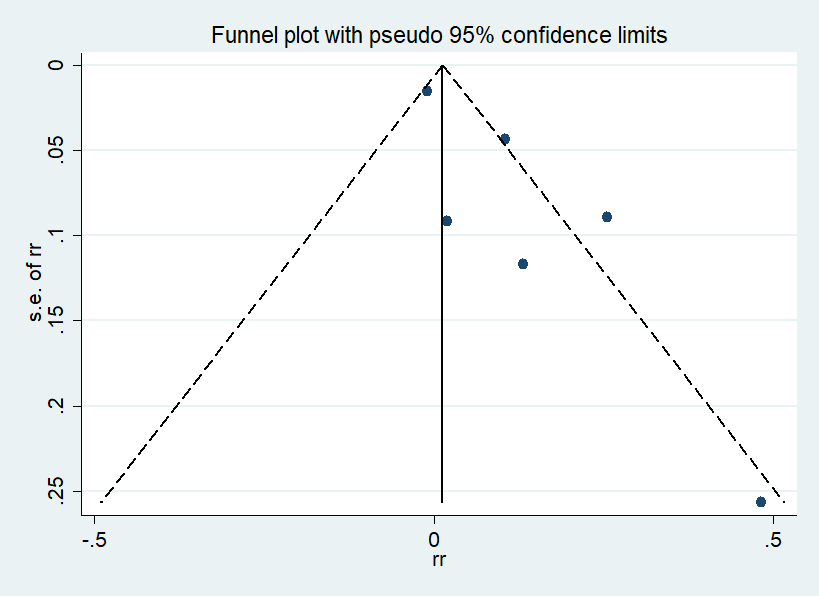

Supplement: Supplementary file 2 [file Image_1.TIF]

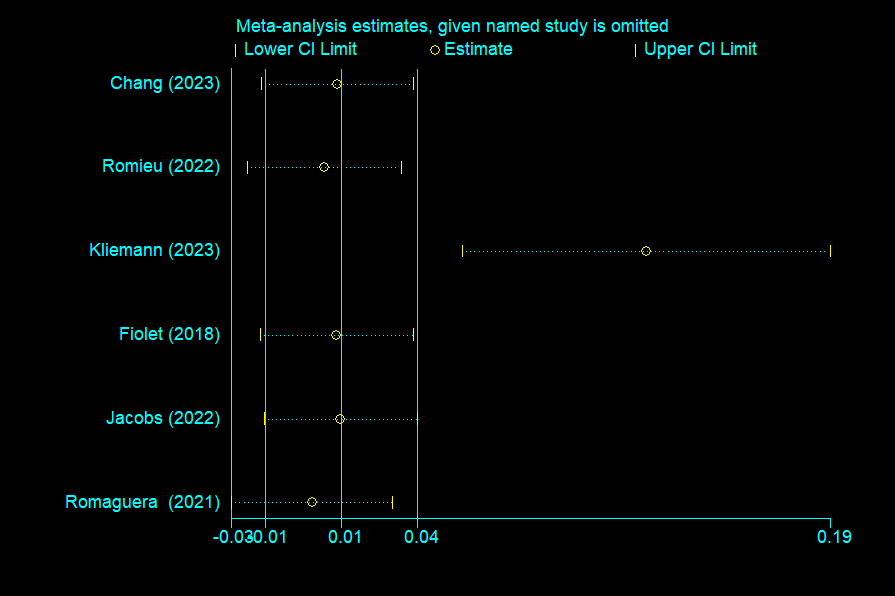

Supplement: Supplementary file 3 [file Image_2.TIF]

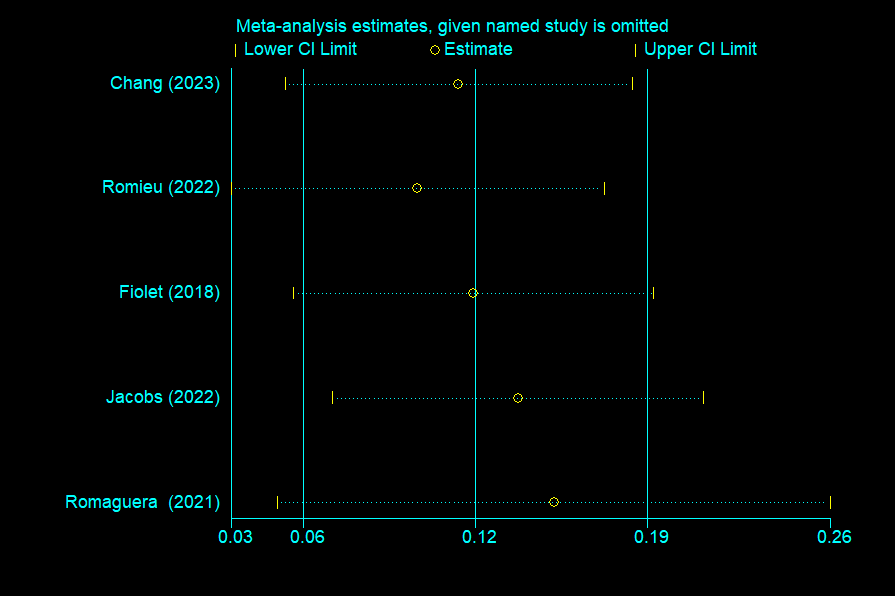

Supplement: Supplementary file 4 [file Image_3.TIF]
